# PREPARING THE CARE WORKFORCE TO ADDRESS INTERPERSONAL DEMANDS ACROSS THE DEMENTIA TRAJECTORY

**DOI:** 10.1093/geroni/igae098.1518

**Published:** 2024-12-31

**Authors:** Scott Trudeau, Laura Gitlin

**Affiliations:** Chair; Discussant

## Abstract

This symposium posits that student learning experiences in professional programs are a direct pipeline to impact workforce readiness to address the needs of people living with dementia (PLWD). The presentations will share data and experience with three innovative learning activities that have been implemented with graduate students in occupational therapy. PLWD often face social isolation due to declines in domains such as memory and communication. This estrangement can extend to the care team, as cognitive declines can lead care professionals to focus increasingly on caregivers. While partnering with caregivers is often essential to optimize the health and well-being of the dyad, this may have the unintended consequence of minimizing the voice and perspective of the person with dementia. Similarly, in institutional settings, custodial care may be prioritized over attending to quality-of-life due to staffing challenges and a lack of creativity regarding person-centered practice for those with later stage dementia. These abstracts present three programs, generated to address identified care needs, focused on increasing person-centered approaches for PLWD. First, students engaged in preventive approaches to proactively increase the voice of older adults using the My Life Profile. Second, remote delivery of the My Life, My Story (MLMS) program at Department of Veterans Affairs allowed community-dwelling Veterans with cognitive concerns to share their stories with the care team. And lastly, The Reading Buddies program for individuals in later staged dementia will be shared. Providing experiential learning opportunities empowering care workers is critical to improving the future of dementia services across the continuum.

